# Point-of-care ultrasound (POCUS) in Norwegian general practice

**DOI:** 10.1080/02813432.2020.1753385

**Published:** 2020-04-21

**Authors:** Hans-Christian Myklestul, Trygve Skonnord, Mette Brekke

**Affiliations:** a General Practice Research Unit (AFE), Department of General Practice, Institute of Health and Society, University of Oslo, Oslo, Norway;; b Department of General Practice, Institute of Health and Society, University of Oslo, Oslo, Norway

**Keywords:** Point-of-care ultrasound, general practice, gender difference, geographical difference, reimbursement

## Abstract

**Objective:** To assess the use of point-of-care ultrasound (POCUS) in Norwegian general practice.

**Design:** Retrospective register study based on general practitioners’ (GPs’) reimbursement claims.

**Setting:** Norwegian general practice excluding out-of-hours clinics in 2009, 2012 and 2016.

**Subjects:** GPs who scanned patients for a given set of symptoms and medical conditions.

**Main outcome measures:** Number and characteristics of GPs performing POCUS. Number and type of scans carried out.

**Results:** The number of scanning GPs increased from 479 in 2009 to 2078 in 2016. The number of registered scans increased from 8962 to 55921. In 2016, approximately 30% of Norwegian GPs sent at least one reimbursement claim for POCUS. Seven out of 10 GPs did not scan every month. The gender distribution of scanning GPs was equal to that of the total GP population. Male GPs scanned four times more frequent than female GPs. Specialist in family medicine scanned twice as much as non-specialist. The use of POCUS among GPs in different counties varied from 31.6 to 198.5 per 10,000 citizens.

**Conclusions:** The number of Norwegian GPs using POCUS and the number of scans have increased substantially from 2009 to 2016. The use of the various scans, based on the use of reimbursement claims, have evolved differently. The reasons for this are not known. The low number of scans carried out by most GPs raises a concern when it comes to the quality of the performed scans.

**KEY POINTS**
- 30% of Norwegian general practitioners (GPs) used point-of-care ultrasound (POCUS) in 2016.
- The use of POCUS increased six-fold from 2009 to 2016.
- Three out of four scanning GPs performed less than 10 scans annually.
- Male GPs performed 80% of the claimed scans.

## Introduction

Ultrasound has been a part of medical diagnostics since the 1950s. In Norway, a few general practitioners (GPs) have used ultrasound over the last 40 years for a variety of clinical problems [1,2]. As ultrasound devices have evolved, the term point-of-care ultrasound (POCUS) has become established in the field of general practice and became a major trend after 2000 [3–9]. POCUS is a focused examination of patient symptoms as a part of the diagnostic process that includes anamnesis and physical- and laboratory examinations, and is less comprehensive than a full diagnostic ultrasound by radiologists. It is considered safe for GPs to perform POCUS for conditions of low to moderate complexity [3,6,7,10–13].

Norwegian general practice is partially funded from The Norwegian Health Economics Administration (Helfo) based on procedures performed. Since 2007, GPs have had the opportunity to claim reimbursements for certain ultrasound scans [14]. Assessment of residual urine and evaluation of fetal head position at term were the first two procedures covered. From 2009, first-trimester bleeding, deep venous thrombosis (DVT), diseases of gallbladder or aorta, such as abdominal aortic aneurysms (AAA), and pathological skin-associated processes, such as abscesses, were included to the list of allowed procedures [15].

Several previous studies have addressed various aspects of POCUS in general practice, including assessing its use in out-of-hours primary health care [16], and education [4–6,8,17], measuring quality of scanning by GPs [4–8,11,12,17,18], the number of scans in various countries [4,8,16] and financial aspects [4–6,8,16–18]. No study has addressed the development of scanning in general practice for the entire population of GPs in a country. The scope of this study was to investigate Norwegian GPs’ use of POCUS during day-time hours after the introduction of reimbursements. Our research questions were:

1. How many GPs used POCUS, and what characterized these GPs?
2. Which scans did they perform, and how frequently?
3. How has use of POCUS developed?

## Materials and methods

This was a retrospective register study. Datasets including reimbursement claims for POCUS from all Norwegian GPs for 2009, 2012 and 2016 were obtained from Helfo. The variables given were pseudo-ID, age, gender, county of residence and practice and whether the GP was specialist in family medicine or not, for each GP. In addition, reimbursement codes [15] and number of claims were available for each GP. The dataset is thought to be complete. Reimbursement claims without codes for POCUS were not available for this study. The use of POCUS in out-of-hours practices and for POCUS performed by GPs outside the regular GP scheme were not included in the dataset. Patient data were not available for this study.

### Statistics and ethics

Statistical analysis was performed by IBM SPSS^®^ v. 25 using simple descriptive analyses.

The Regional Committee for Medical and Health Research Ethics assessed the project not in need of their approval (ref 2018/213). The Norwegian Directorate of Health assessed the data to be anonymous, thus no need of dispensation from requirement of professional secrecy (ref 18/142-2).

## Results

We identified 2675 unique GPs, 1627 male and 1048 female, who claimed reimbursement for the use of POCUS over the three study years. For 2009, 2012 and 2016 there were 479, 953 and 2078 unique GPs, respectively. These numbers included list-holding GPs, interns and locums. At the end of 2016, there were 4667 GPs in Norway [19]. There were 1350 interns each year, and including these and locums [19], the percentage of scanning GPs in 2016 was approximately 30%. For the 2009 and 2012, the numbers were 8.5 and 16 percent, respectively. There was a six-fold increase in the number of scans from 8962 in 2009 to 54,931 in 2016. In total, 101,428 reimbursement claims for POCUS were made over the three years. Female GPs increased the number of claims from 1390 in 2009 to 10913. There was an observed difference in the use of POCUS between age-cohorts. GPs aged 25 through 35 reported a four-fold increase from 1038 in 2009 to 4152 in 2016. GPs 66 and older had a more than 14-fold increase from 346 reimbursement claims in 2009 to 4953 in 2016.

**Figure 1.**
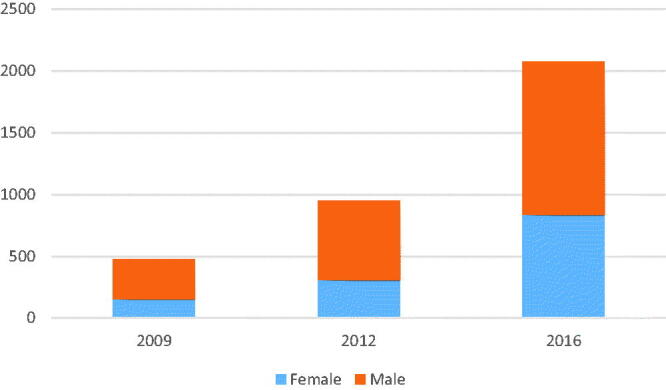
Number of GPs claiming reimbursement for POCUS, by gender.

Almost 40% of GPs who claimed reimbursement were women (Figure 1). Male GPs made 82,299 claims, more than 81% of the total. Among the various scans, these proportions varied. For skin-near pathological processes, male GPs scanned 18,029 times in 2016, female GPs 2753, a more than six-fold difference. For fetal head-position at term there were in 2016 3263 male GP scans and 1552 female GP scans. For first trimester-bleeding, there was a similar tendency with 2461 scans performed by male GPs and 1774 by female GPs. For the other scans, the 4:1 proportion held true. There was no difference between specialists and non-specialists in number of scanning GPs. Specialists made 68,466 of the registered claims covering 68% of all reported scans in the period.

**Figure 2.**
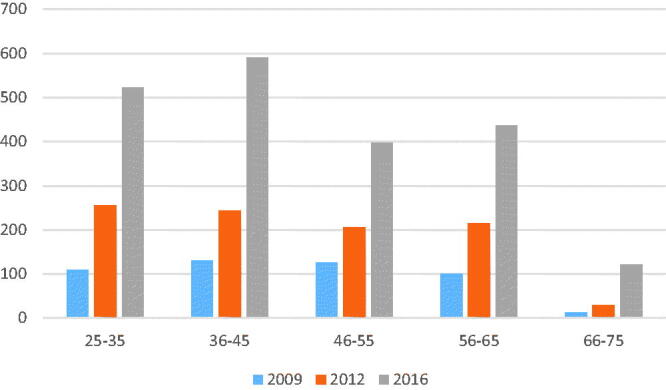
Number of GPs claiming reimbursement for POCUS, by age.

The increase over time in number of scanning GPs of different age groups was equal, although amongst GPs older than 66 years the number doubled from 2009 to 2016 (Figure 2). Approximately 6% of scanning GPs were age 66 years or more, whereas this age group counted for 3.6% of the GP population [19]. For the various scans, the use of POCUS evolved differently in different age cohorts. GPs aged 56 through 65 increased the number of scans for skin-associated processes from 163 in 2009 to 6660 in 2016. For GPs aged 25 through 35, there was only an increase from 126 to 1381. Scans for diseases of the gallbladder or aorta increased amongst GPs older than 65 years from 104 in 2009 to 2239 in 2016. The assessment of residual urine differed between two of the cohorts. GPs aged 25 through 35 had almost no increase in their use of POCUS for this procedure: from 478 in 2009 to 647 in 2016. GPs aged 66 or more had an increase from 48 to 594 in the same period.

**Figure 3.**
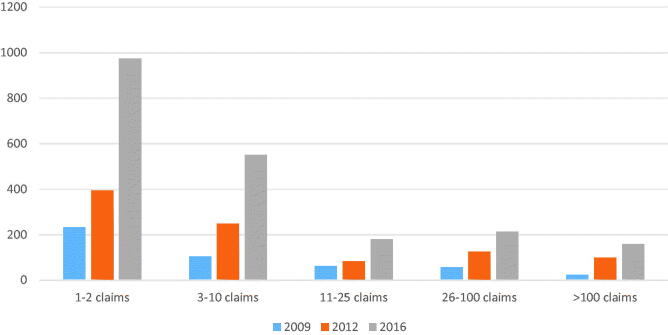
Number of GPs grouped after number of POCUS reimbursement claims per year.

The mean number of scans per year per included GP was 37.9. The distribution of the number of claims was wide, with the highest number being a total of 2658 reported scans over the three years. Figure 3 shows the distribution of GPs according to their number of claims. There was an increase in claims for reimbursement in all groups of GPs, but the highest increases were in the two groups of the fewest claims. GPs scanning one or two times per year accounted for half of the scanning GPs, but did only make two percent of all performed scans. There was a difference in type of performed scans between the GPs making one or two scans annually and the rest. The biggest difference was observed in pathological skin-near processes, where the frequent scanners proportionally scanned five times as often as compared to the low scanning group. Opposite, the low-scanning group scanned for DVTs three times as often and almost twice as often for diseases of aorta and gallbladder compared to the total numbers of scans, as did the frequent scanning GPs. For the other scans, there were no differences.

**Figure 4.**
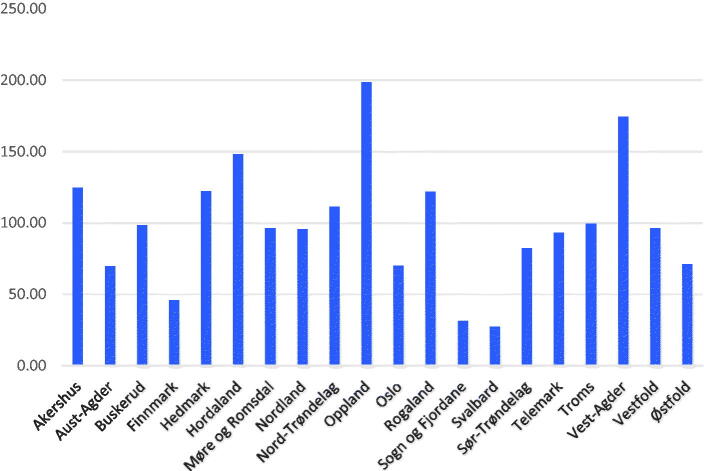
Numbers of ultrasound reimbursements claims per 10,000 citizens by Norwegians counties.

Figure 4 presents the distributions of claims in the various counties of Norway for 2016. The number of scans per 10,000 citizen varied from 31.6 to 198.5. The distribution was similar for 2009 and 2012.

**Figure 5.**
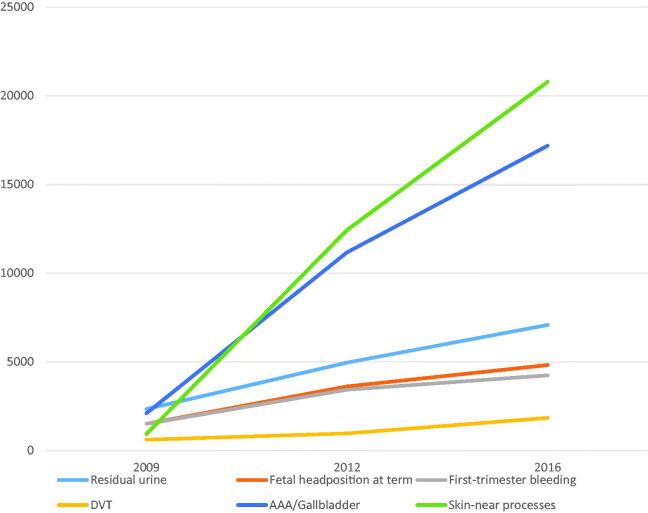
Number of POCUS reimbursements claims by type of examination.

Figure 5 shows the changes in number of claims for each reimbursement code. Claims for scanning of residual urine, fetal head position at term, DVT and first-trimester bleeding increased three-fold from 2009 to 2016. In the same period, number of claims for diseases of gall bladder or aorta increased eight-fold. The greatest increase in claims was observed for skin-associated processes such as abscesses, which showed a 22-fold increase in claims from 932 in 2009 to 20,782 in 2016.

## Discussion

From 2009 to 2016, the number of Norwegian GPs using POCUS increased four-fold. The gender distribution equaled that of the total GP population. However, male GPs scanned four times as often as female GPs in term of number of scans. Specialist status in family medicine did not affect the number of scanning GPs, however specialists made more than twice as many claims as non-specialists. Variation in the use of POCUS between counties was more than six-fold. The use of POCUS for various scans increased from three-fold to 22-fold.

Our data found that approximately 30% of GPs claimed the use of POCUS in 2016. Although previous studies have looked at several aspects of GPs’ use of POCUS [4,5,8], no study has addressed the frequency of POCUS use in an entire GP population. There is a long history of GPs using POCUS in Norwegian general practice [1,2,20,21], partially explained by long distances to the nearest hospital [2,21]. Our findings of a four-fold increase in scanning GPs from 2009 to 2016 may be explained by reduced costs for the ultrasound devices [13] and their improved ease of use [7,11,13,17]. In addition, there has been a nationwide tendency of single-handed GP practices to combine into larger clinics, making an investment in such equipment more reasonable. Increased availability of POCUS in these clinics may have encouraged colleagues to scan [22]. The introduction of reimbursement claims in 2007 may also have contributed to the increased use of POCUS. However, to break even financially, a Norwegian GP must currently scan and claim reimbursement twice daily (personal data). Our findings do not suggest such practice, as can be seen in Figure 3.

The 40% of GPs claiming reimbursement were women (Figure 1) and was proportionate to the 42% of Norwegian GPs who were women [19]. There was no observed gender difference between GPs in terms of number of scanning GPs. These results differ from the results described by Myhr et al. in out-of-hours clinics [16], where 74% of scanning GPs were men. The difference may be explained by more male GPs working out-of-hours. In terms of number of performed scans, male GPs used POCUS more, as 81% of the performed scans was made by male GPs. This tendency for increased use of imaging techniques among male GPs has been shown previously by Ringberg et al. [23].

Data from 2011 to 2013 showed that 16.7% of Norwegian general practices had ultrasound devices available [24]. We observed 16% of Norwegian GPs claiming reimbursement for 2012, which confirms the first observation. A study of Norwegian out-of-hours practices in 2016 reported that 23% of practices had POCUS available [16], with 6.5% of physicians scanning in comparison to 30% during office hours. The discrepancies in scanning among physicians between regular office hours and out-of-hours practice cannot be explained by our study. Limited time available, poorer knowledge of patients and different devices are possible causes.

According to our data, seven out of ten GPs using POCUS made 10 or fewer scans annually. However, the number of GPs who performed more than 100 scans per year increased almost seven-fold over the study period. The smallest increase was found in the group performing 11–25 scans annually, which increased less than three-fold. The low frequency of scans for most GPs raises a concern about the quality of the performed scans. Ultrasonography is considered to be a highly user-dependent clinical skill [8,17,18]. However, for several types of scans, such as those to detect an AAA, even novices can scan with a high level of diagnostic accuracy [3,6,7,10–13]. There may be many causes to the low number of scans among the majority of GPs. First, an average GP can expect to see conditions such as DVT [25] or first trimester bleeding only a few times a year. Availability of the equipment is another aspect that may affect the use of POCUS. Having your own ultrasound apparatus in stand-by mode next to the patient makes scanning more feasible compared to if you have to go and get it in another room. On the other hand, our study does not indicate that the GPs, the majority of whom are private practitioners, used POCUS claims to increase their income, see Figure 3.

POCUS has been integrated as a part of the curriculum in medical studies in several universities [4,26,27] and has also been included in some family medicine residence programs [4,8,27]. We did not expect to observe the effect of this in Norway during the study period. The relatively high quality of scans for simpler assessments, even by novices, may also have contributed to the increased number of scans [3,6,7,10–13].

The oldest cohort of GPs had the highest increase in the use of POCUS over the period. The explanation may be better economy and perhaps more time to follow diagnostic and therapeutic curiosity. Experienced GPs have been in practice for a longer time, and may see older patients in whom diseases are more likely to occur. The increase in performed scans for residual urine and AAA may support this hypothesis. The lack of difference between specialists and non-specialists in number of scanning GPs is consistent with 57% of GPs who were specialists in family medicine [19]. The proportionate increase in scanning specialists and non-specialist was presumably partly due to an adaptation of younger GPs to the technological advances in primary care. The higher proportion of specialists claiming reimbursement may be explained by the likelihood of experienced GPs being specialist.

Norwegian geography and long distances to the nearest hospital have been used as arguments to advocate the use of POCUS in general practice [2,21]. Our data do only partially support this, as shown in Figure 4. The observed dispersion of use of POCUS was not correlated with geography or locations of hospitals in the various counties. Local enthusiasts and traditions may have contributed to the local variations [1,2,21,28,29].

The extent of POCUS for various organ examinations evolved differently. The three-fold increase for residual urine, fetal head position at term, first-trimester bleeding and DVT was lower than the increase in number of scanning GPs. The eight-fold increase in scanning for gallstones or AAA can partially be explained by their ease in performing [3,6,7,10–13] and clinical significance. The 22-fold increase in scanning for skin-associated processes cannot be explained and requires further studies.

## Limitations

The use of ultrasound in general practice is broader than the scans included in this study [1,4,8,30]. For example, GPs use ultrasound for diagnosis and treatment of musculoskeletal disorders, but because such use did not lead to a reimbursement claim, these scans were not counted in our study. Bladder scanning performed by other healthcare professionals is a source of error that cannot be accounted for in these numbers. Both the number GPs using POCUS and the number of scans for residual urine can be lower than those registered. Reimbursement claims for first-trimester bleeding, DVT, diseases of gallbladder and aorta and pathologic processes close to the skin were only instituted in the second half of 2009, so the scans recorded for these procedures were all performed in the second half of that year. Another source of error is the fact that the register is based on self-reported claims of procedures performed. There was a possibility that GPs forgot to code for all performed procedures. The possibility of reporting more performed procedures than those who were actually performed is also present.

The numbers of locums were probably higher for all three registered years. Until the second half of 2016, only vacancies lasting for more than two months were registered [19]. In Norway, interns rotated to general practice in March and September. Although there were approximately 1350 different interns in total, only one-third of these were in the clinics at the same time. This causes some uncertainty about the interpretation of the percentage of scanning GPs.

## Conclusions

Overall, 30% of Norwegian GPs claimed the use of POCUS in 2016. There was a four-fold increase in number of scanning GPs and six-fold increase in performed scans since 2009. The observed variations in scanning related to GP gender, the types of scans and geographical variations are not easily explained. The limited number of performed scans by the majority of GPs is a source of concern when addressing the quality of the use of POCUS.

## References

[1] Bratland SZ. Ultralyddiagnostikk anvendt i almenpraksis. Samlet vurdering [Ultrasonic diagnosis used in general practice. A summarized evaluation]. Tidsskr nor Laegeforen. 1985;105:1954–1955. 3907006

[2] Eggebo TM, Dalaker K. Ultralydundersøkelser av gravide i allmennpraksis [Ultrasonic diagnosis of pregnant women performed in general practice]. Tidsskr nor Laegeforen. 1989;109:2979–2981. 2686093

[3] Hall JW, Holman H, Bornemann P, et al. Point of care ultrasound in family medicine residency programs: a CERA study. Fam Med. 2015;47(9):706–711. 26473563

[4] Mengel-Jorgensen T, Jensen MB. Variation in the use of point-of-care ultrasound in general practice in various European countries. Results of a survey among experts. Eur J Gen Pract. 2016;22(4):274–277. 27487159 10.1080/13814788.2016.1211105

[5] Genc A, Ryk M, Suwała M, et al. Ultrasound imaging in the general practitioner’s office - a literature review. J Ultrason. 2016;16(64):78–86. 27104005 10.15557/JoU.2016.0008 PMC4834373

[6] Nelson BP, Sanghvi A. Out of hospital point of care ultrasound: current use models and future directions. Eur J Trauma Emerg Surg. 2016;42(2):139–150. 26038015 10.1007/s00068-015-0494-z

[7] Bornemann P, Barreto T. Point-of-care ultrasonography in family medicine. Am Fam Physician. 2018;98(4):200–202. 30215979

[8] Andersen CA, Holden S, Vela J, et al. Point-of-care ultrasound in general practice: a systematic review. Ann Fam Med. 2019;17(1):61–69. 30670398 10.1370/afm.2330 PMC6342599

[9] Moore CL, Copel JA. Point-of-care ultrasonography. N Engl J Med. 2011;364(8):749–757. 21345104 10.1056/NEJMra0909487

[10] Lindgaard K, Riisgaard L. Validation of ultrasound examinations performed by general practitioners. Scand J Prim Health Care. 2017;35(3):256–261. 28776457 10.1080/02813432.2017.1358437 PMC5592352

[11] Andersen GN, Viset A, Mjolstad OC, et al. Feasibility and accuracy of point-of-care pocket-size ultrasonography performed by medical students. BMC Med Educ. 2014;14(1):156. 25070529 10.1186/1472-6920-14-156 PMC4131775

[12] Diprose W, Verster F, Schauer C. Re-examining physical findings with point-of-care ultrasound: a narrative review. N Z Med J. 2017;130(1449):46–51. 28178729

[13] Wittenberg M. Will ultrasound scanners replace the stethoscope? BMJ. 2014;348(7):g3463–g3463. 24875141 10.1136/bmj.g3463

[14] Kristoffersen JE, Roksund G. Ultralyd i allmennpraksis [Ultrasonography in general practice]. Tidsskr nor Laegeforen. 2007;127:2414. 17895954

[15] Lovdata. Forskrift om stønad til dekning av utgifter til undersøkelse og behandling hos lege [Regulations for grants to cover the costs of examination and treatment by a doctor]: Lovdata; 1981. [cited 2018 Sep 07]. Available from: https://lovdata.no/dokument/SF/forskrift/2018-06-29-1153.

[16] Myhr K, Sandvik H, Morken T, et al. Point-of-care ultrasonography in Norwegian out-of-hours primary health care. Scand J Prim Health Care. 2017;35(2):120–125. 28593825 10.1080/02813432.2017.1333307 PMC5499311

[17] Laerum F, Mørland B. Ultralyddiagnostikk i primaerhelsetjenesten – ny teknologi kan gi økt utbredelse [Ultrasounddiagnostics in primary health care - new technology can cause extensive use]. Tidsskr nor Legeforen. 2001;121:3101–3103. 11757447

[18] Wordsworth S, Scott A. Ultrasound scanning by general practitioners: is it worthwhile?. J Public Health Med. 2002;24(2):88–94. 12141591 10.1093/pubmed/24.2.88

[19] Management data for the GP scheme 2016 Oslo, Norway: Norwegian Directorate of Health; 2016. [cited 2017]. Available from: https://www.helsedirektoratet.no/statistikk/statistikk/fastlegestatistikk/hovedtallsrapport%20fastlegeordningen%20landstall%202016.pdf/_/attachment/inline/f5fa67f1-7254-4094-bd24-11b757483e42:e5d70aa7b8858dc69274bf1656cf93671037b28f/hovedtallsrapport%20fastlegeordningen%20landstall%202016.pdf.

[20] Bratland SZ, Ødegaard S. Ultralydundersøkelse – noe for allmennpraksis? [Ultrasonography – something for general practice?]. Tidsskr nor Legeforen. 2007;127:1923. 17700730

[21] Nilsen NR. Ultralyd i allmennpraksis [Ultrasonography in general practice]. Tidsskr nor Legeforen. 2001;121:3444. 11826795

[22] Gilja OH. Mobilultralyd i en medisinsk avdeling [Mobile ultrasounddevice in a medical ward]. Tidsskr nor Legeforen. 2003;123:2713–2714. 14600742

[23] Ringberg U, Fleten N, Deraas TS, et al. High referral rates to secondary care by general practitioners in Norway are associated with GPs’ gender and specialist qualifications in family medicine, a study of 4350 consultations. BMC Health Serv Res. 2013;13(1):147. 23617296 10.1186/1472-6963-13-147 PMC3640906

[24] Eide TB, Straand J, Bjorkelund C, et al. Differences in medical services in Nordic general practice: a comparative survey from the QUALICOPC study. Scand J Prim Health Care. 2017;35(2):153–161. 28613127 10.1080/02813432.2017.1333323 PMC5499315

[25] Olaf M, Cooney R. Deep venous thrombosis. Emerg Med Clin North Am. 2017;35(4):743–770. 28987427 10.1016/j.emc.2017.06.003

[26] Geitung JT, Grøttum P. Ultralyd som integrert del av medisinstudiet [Ultrasonography as an integrated part of the medical curriculum]. Tidsskriftet. 2016;136(14/15):1192–1192. 10.4045/tidsskr.16.0544 27554553

[27] Micks T, Braganza D, Peng S, et al. Canadian national survey of point-of-care ultrasound training in family medicine residency programs. Can Fam Physician. 2018;64(10):e462–e467. 30315038 PMC6184970

[28] Glaso M, Medias IB, Straand J. Diagnostisk ultralyd i en fastlegepraksis [Diagnostic ultrasound in general practice]. Tidsskr nor Laegeforen. 2007;127:1924–1927. 17700731

[29] Borthne R, Lied A, Karevold A. Bobling i brystet [Gurgling in the chest]. Tidsskriftet. 2014;134(1):47–47. 10.4045/tidsskr.13.1054 24429756

[30] Lokkegaard T, Todsen T, Nayahangan LJ, et al. Point-of-care ultrasound for general practitioners: a systematic needs assessment. Scand J Prim Health Care. 2020;38 (1):1–9. 31955658 10.1080/02813432.2020.1711572 PMC7054965

